# Effect of the Enrichment in c-Kit Stem Cell Potential of Foetal Human Amniotic Fluid Cells: Characterization from Single Cell Analysis to the Secretome Content

**DOI:** 10.3390/biomedicines11020430

**Published:** 2023-02-02

**Authors:** Francesca Casciaro, Francesca Beretti, Martina Gatti, Giuseppe Persico, Emma Bertucci, Marco Giorgio, Tullia Maraldi

**Affiliations:** 1 Department of Biomedical Sciences, University of Padua, 35131 Padova, Italy; 2 Department of Biomedical, Metabolic and Neural Sciences, University of Modena and Reggio Emilia, 41124 Modena, Italy; 3 Department of Experimental Oncology, IRCCS–European Institute of Oncology, 20139 Milano, Italy; 4 Department of Medical and Surgical Sciences for Mothers, Children and Adults, University of Modena and Reggio Emilia, Azienda Ospedaliero Universitaria Policlinico, 41124 Modena, Italy

**Keywords:** sorting, amniotic fluid stem cells, transcriptome, stemness

## Abstract

Human amniotic fluid cells (hAFSCs) are a fascinating foetal cell-type that have important stem cell characteristics; however, they are a heterogeneous population that ranges from totally differentiated or progenitor cells to highly multipotent stem cells. There is no single approach to isolating the stem cell component, but the selection of a subpopulation of hAFSCs expressing c-Kit is widely employed, while a deep characterization of the two populations is still lacking. Here we performed single-cell and bulk RNAseq analysis to compare the gene expression profiles of adherent amniotic fluid cells and their subpopulation c-Kit^+^. Information deriving from this high throughput technology on the transcriptome was then confirmed for specific targets with protein expression experiments and functional analysis. In particular, transcriptome profiling identified changes in cellular distribution among the different clusters that correlated with significant differential expression in pathways related to stemness, proliferation, and cell cycle checkpoints. These differences were validated by RT-PCR, immunofluorescence, WB, and cell cycle assays. Interestingly, the two populations produced secretomes with different immune-modulating and pro-regenerative potentials. Indeed, the presence of TGFβ, HGF, IDO was higher in EVs deriving from c-Kit^+^ cells, unlike IL-6. These results suggest the existence of deep intra-population differences that can influence the stemness profile of hAFSCs. This study represents a proof-of-concept of the importance of selecting c-Kit positive fractions with higher potential in regenerative medicine applications.

## 1. Introduction

In the last two decades, amniotic fluid cells have been demonstrated to be an interesting population of foetal cells with unique characteristics that make them distinctive from other sources of stem cells (SCs): indeed, amniotic fluid stem cells (AFSCs) have numerous features, expressing both markers and transcription regulators of pluripotency and mesenchymal commitment, thus many authors have suggested an intermediate state for them, between embryonic stem cells (ESCs) and mesenchymal stromal cells (MSCs), namely called broadly multipotent [1,2,3]. As a matter of fact, besides mesodermal lineage, AFSCs are able to differentiate into the other lineages, endodermal [4] and ectodermal [5].

Even looking only at hAFSCs obtained during the second trimester (from amniocentesis), various classifications have been proposed for human amniotic subpopulations, including a morphological classification, since up to three cell types have been described, from epithelioid to spindle-shaped [6]. Therefore, amniocytes differ in many functional aspects, including survival rate, plastic adhesion, and growth characteristics. Regarding the expression of stem cells markers, it seems that the core pluripotency markers, Oct4, Nanog, and SOX2, are expressed only in specific cells that contain a small percentage of amniocytes in primary cultures, and that these target genes exhibit heterogeneity and variability in hAFSC samples. This difference can be due to the diverse derivation site of amniocytes and to their individual genetic backgrounds, as demonstrated by several authors [7,8].

Moreover, amniotic fluid (AF) contains numerous terminally differentiated cells and progenitor cells with more limited differentiation potentials. In order to limit this variability and to obtain the subpopulation with the highest stem characteristics, since 2007 hAFSCs expressing c-Kit (or CD117), a surface antigen of the type III tyrosine kinase identified as a stem cells marker, have been isolated in several labs by an immunoselection approach using magnetic microspheres [9]. They also displayed expression of pluripotency markers, including Oct4, Nanog, and SOX2. In this regard, Moschidou and colleagues revealed that c-Kit^+^ hAFSCs have 82% transcriptome identity with ESCs, in addition to the capability of forming embryoid bodies in vitro [9]. However, the percentage of c-Kit positive cells is variable and weak, from 1 to 5% of total adhering cells. Once sorted for c-Kit, hAFSCs have to be expanded again in order obtain a sufficient number to be used for experimental and clinical applications, but many studies have shown a remarkable reduction in the expression of the key pluripotency markers of Oct4, Nanog, and SOX2 throughout the long-term culture of different human MSCs [10]. Therefore, it is of the highest relevance to precisely investigate the advantages and possible drawbacks of isolating the c-Kit positive subpopulation of amniotic fluid cells. Regarding other stem cell types, it has been demonstrated that the enrichment in c-Kit positive cells contributes to the preservation of the self-renewal capacity of dental pulp stem cells [11] and an undifferentiated state for bone marrow stem cells [12] and improves the differentiation potential for placental cells [13,14].

Regarding amniotic fluid cells, Bai and co-workers in 2012 [15] compared human AF-derived c-Kit^+^ stem cells with the c-Kit^−^ population, demonstrating that both types of AFS cells could differentiate along adipogenic and osteogenic lineages, while the myocardial differentiation capacity was enhanced in c-Kit^+^ AFS cells. Their RT-PCR and immuno-cytochemical analysis confirmed the flow cytometry results that the c-Kit^+^ AFS cells showed strong *Oct4*, *SOX2*, and *Nanog* expression, and that the c-Kit^−^ AFS cells did not express these genes. While this is an encouraging study, it did not provide a comparison between the two processes of isolation (with or without selection), but rather between the c-Kit-expressing population and the one depleted to c-Kit^+^ cells.

A comprehensive study describing the differences between c-Kit^+^ cells and the total population of adhering amniotic fluid cells is still lacking. In this study, we analysed with different approaches these cell populations, starting from new high throughput assays to classical cell biology experiments on the cells and their secretome in order to drive this unique cellular model towards more clinical applications.

## 2. Results

### 2.1. Transcriptomic Profiles of the Two Different hAFSCs Populations

**Figure 1:**
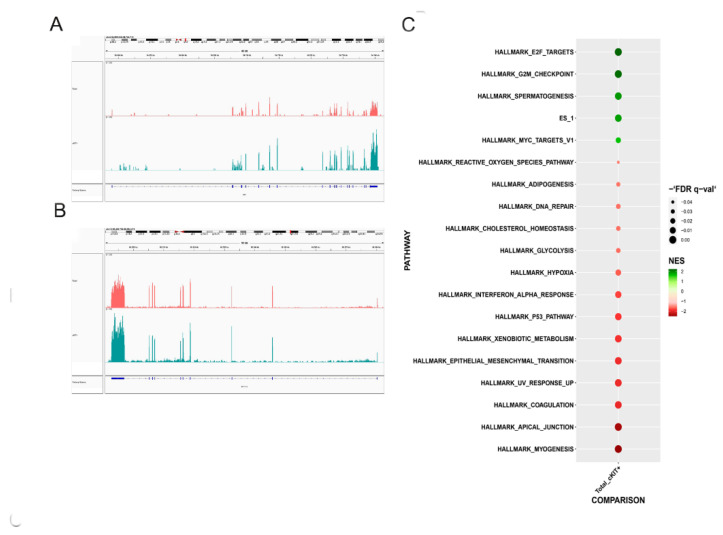
Total amniotic fluid cells and selected for c-Kit: analysis with bulk-RNAseq. (**A**) Gene expression level of *KIT* gene in the total (purple) and the selected sample (blue). (**B**) Gene expression level of *KIT ligand* gene in the total (purple) and the selected sample. (**C**) Gene set enrichment analysis using HALLMARK collection. Upregulated pathways in selected cells are in green, while downregulated pathways are in red.

In our previous study, we proposed a method to isolate a subpopulation of c-Kit positive cells with the highest stemness properties, demonstrating that also at a transcriptome level [7]. To investigate how the selection affects samples’ heterogeneity and to understand if the process tends to select populations with peculiar characteristics, we decided to perform a single cell RNA analysis experiment. Prior to that, to be sure that we were operating on selected cells and under conditions similar to those observed in our previous project, we analysed the transcriptome in bulk to gain an idea of the average expression level of the genes in our samples. *KIT* gene was higher expressed in the selected sample (Figure 1A), confirming the bona fide of the selection process, and its ligand, the stem cell factor (SCF), was higher expressed as well (Figure 1B). Then, we decided to perform a gene set enrichment analysis, which revealed results similar to those obtained in our previous work, namely, that c-Kit enrichment induced an increase of pathways related to stemness (ES1), proliferation (E2F), and cell cycle checkpoints (G2M). On the other hand, selected cells showed, among others, a downregulation of the epithelial-mesenchymal transition pathway and, to a lower extent, of the reactive oxygen species pathway. Regarding this pathway, we can notice that the expression of many antioxidant enzymes was differently regulated in the two samples (Supplementary Figure S1). A controversial result, with respect to our previous publication, was obtained for hypoxia, which was in this case downregulated (Figure 1C).

**Figure 2:**
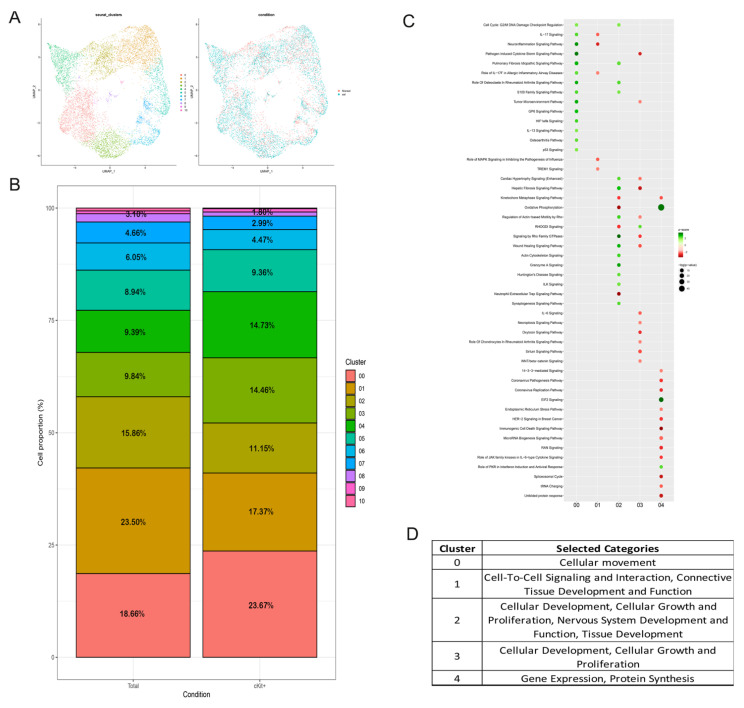
Total amniotic fluid cells and selected for c-Kit: comparison of the transcriptomic profile with single cell analysis. (**A**) UMAP plot displaying the number of identified hAFSC clusters and the distribution of cells belonging to the two different conditions. (**B**) Percentage of cells in each identified cluster with respect to the total number of purified cells in the total (on the left) and c-Kit^+^ sample (on the right). (**C**) Ingenuity pathway analysis performed on the class-specific markers of the five most abundant clusters: upregulated pathways in c-Kit^+^ cells are in green, while downregulated pathways are in red. (**D**) Selected categories identified with IPA analysis using specific markers of the first five most abundant clusters.

At this point, we were ready to perform the single cell analysis, which revealed the presence of 11 distinguishable clusters in both the selected and the non-selected samples (Figure 2A). Even if the selection process seemed not to affect the number of clusters, a different distribution of cells within them was observed. In particular, focusing on the first six and most abundant clusters, selected cells were more abundant in cluster 0, 3, 4, while their number was reduced in cluster 1 and 2 compared to the total population (c-Kit^+^ and total) (Figure 2B). 

To figure out the characteristics of these cells, we identified cluster-specific markers and used them to run an Ingenuity Pathway Analysis. Among pathways, cluster 0 was characterized by an upregulation of G2/M checkpoint (and also in 2), IL-17 (downregulated in 1), IL-13 signaling, and HIF-1α signaling. Markers of cluster 3 seemed to downregulate IL-6 signaling, while cluster 4 was characterized by a strong upregulation of oxidative phosphorylation (downregulated in cluster 2) and EIF2 signaling (Figure 2C).

To better understand these results and drive our research, we crossed them also with biological functions and, among the numerous terms not always directly related to our topic, we selected very few of them that we considered significant for our study. In particular, cluster 0 seemed to increase the function of cell movement, cluster 1 upregulated functions related to cell-to-cell signaling and tissue development, cluster 2 reported an increase of cellular growth proliferation, cluster 3 presented markers of stem cell proliferation, and lastly cluster 4 was characterized by an increase of gene expression and protein synthesis (Figure 2D).

Having collected all these results, we moved on to a deeper characterization of the phenotype of the selected cells.

### 2.2. Phenotypic Characterization of Human Amniotic Fluid Cell Cultures: Effect of c-Kit Selection

**Figure 3:**
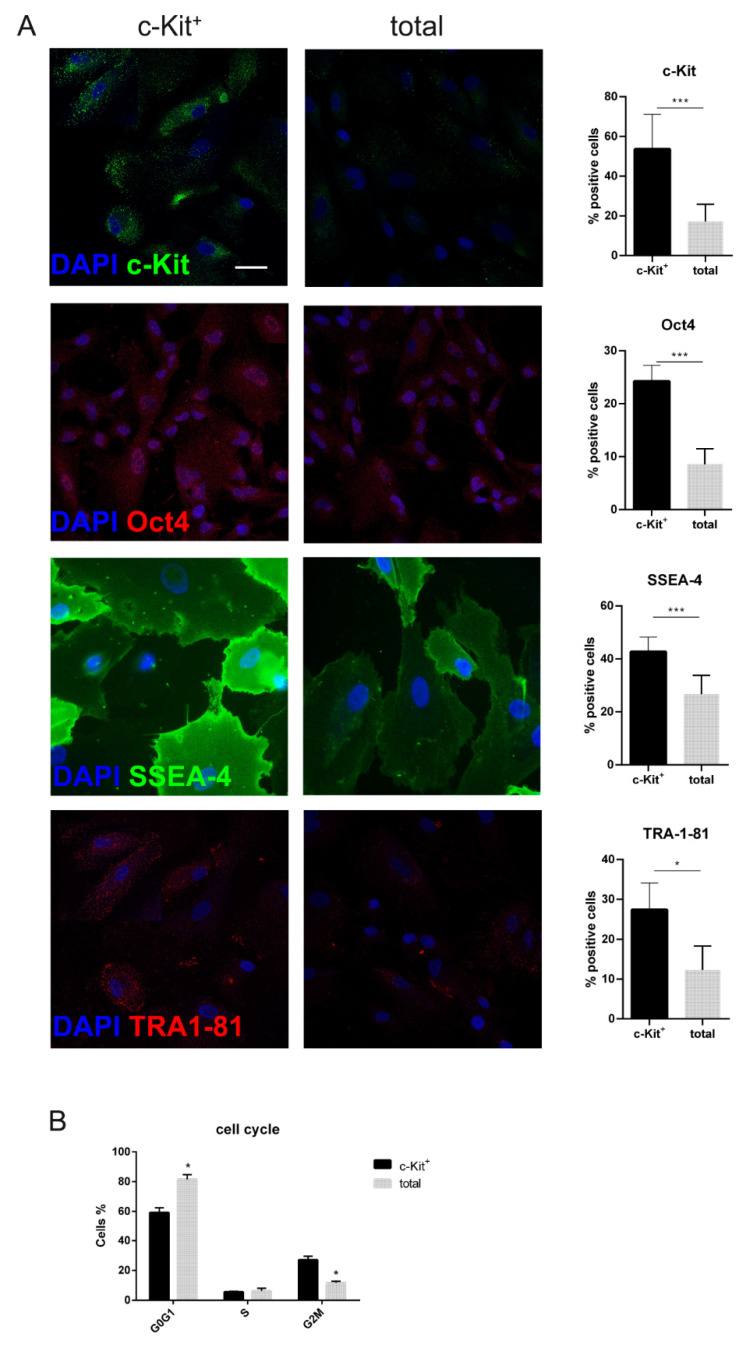
Total amniotic fluid cells and selected for c-Kit: comparison of cell cycle and stemness profile. (**A**) Representative immunofluorescence images and the relative graphs showing the expression of c-Kit, Oct4, SSEA-4, and TRA1-81 in total hAFSCs and c-Kit^+^ cells. Images are superimposed between DAPI (blue) and c-Kit, Oct4 (both in green), SSEA-4 or TRA1-81 (both in red) signals. Scale bar= 10 µm. *** *p* < 0.001 = samples significantly different. (**B**) Quantitative cell cycle analysis by flow cytometry of total hAFSC and c-Kit^+^ samples: * *p* < 0.05 = samples significantly different. At least 10,000 cells were evaluated for each cytofluorimetric analysis.

In order to confirm the selection efficacy and the maintenance of c-Kit after the first passages, immunofluorescence analysis was performed. The comparison showed that the selection process enriches by three times the cell population of c-Kit positive cells. This effect was accompanied by the higher presence of cells positive for other markers of stem cells, such as Oct4, SSEA-4, and TRA1-81 (Figure 3A). 

Interestingly, cell cycle analysis (Figure 3B) clearly showed that, compared to total samples, c-Kit^+^ samples included a higher percentage of cells in G2M at the expense of cells in G0G1, which supports the hypothesis suggested by the bulk and single cell RNA-sequencing.

### 2.3. hAFSC Populations and Their Mitochondrial Phenotype

**Figure 4:**
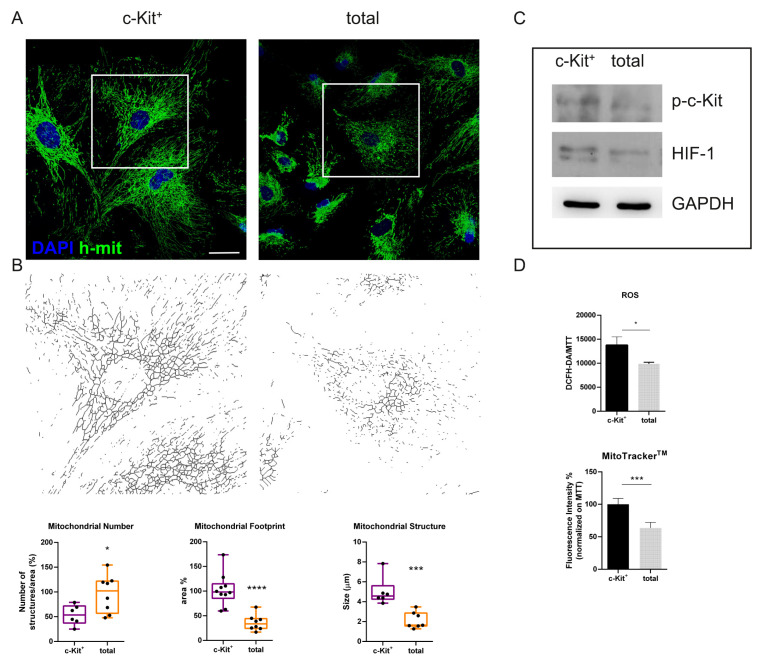
Comparison of mitochondrial morphology and ROS levels. (**A**) Representative immunofluorescence images and the relative graphs showing the morphological pattern (number, size, and footprint) of mitochondria in total hAFSCs and c-Kit^+^ cells. Confocal images are superimposed between DAPI (blue) and h-mit (green). (**B**) MiNA analysis has been performed on the skeletonized images rendering shown below in black and white. Scale bar = 10 µm. **** *p* < 0.0001; *** *p* < 0.001; * *p* < 0.05 = significantly different from c-Kit+ samples. (**C**) Representative images of Western blot analysis of hAFSC samples, revealed with anti-phospho-c-Kit, anti-HIF1α, and anti-GAPDH, as a loading control. (**D**) Representative graphs showing fluorescence, obtained with probes for ROS (DCFH-DA) and mitochondrial signal (MitoTracker), normalized to MTT viability values of hAFSCs samples. *** *p* < 0.001; * *p* < 0.05 = samples significantly different.

The classification of *Ingenuity Pathways Analysis* drove us to investigate the mitochondrial morphology of the two samples. Figure 4A shows representative confocal images of mitochondria: mitochondria of the c-Kit^+^ samples have a more elongated morphology. This observation was supported by image informatic analysis (obtained by MiNA ImageJ-toolset) presented in graphs in Figure 4B, which show the number of mitochondria to be higher in the total samples, while their size, namely length and area, is smaller, compared to c-Kit^+^ samples. This data confirms the modulation in mitochondrial gene expression related to fusion-fission processes observed in single cell analysis where a downregulation of fission genes (*FIS1* and *MFF*) and an upregulation of fusion genes (*MFN1*, *MFN2*, and *OPA1*) was detected in the c-Kit^+^ cells with respect to the total ones along all the clusters (Supplementary Figure S2). Since it is well established that mitochondrial dynamics can be altered by changes in extracellular and/or intracellular stimuli such as reactive oxygen species (ROS) and redox signaling [16], looking at the ROS content shown in Figure 4C we can see that c-Kit^+^ cells have a higher content of total ROS/RNS, as detected by DCFH-DA probe, but the mitochondrial signal is higher in a similar manner, as shown by MitoTracker probe. 

The contribution of the HIF family to the maintenance of SC pluripotency and the link between HIF-1α and ROS to the self-renewal capacity of SCs under normoxic conditions have been surrounded by controversy. WB and ELISA experiments have been performed in order to see the effective protein levels in the two cell samples after some passages in culture. As shown in Figure 4D, the data obtained by single cell analysis was confirmed by WB; moreover, an ELISA test quantified the HIF-1α expression, showing that in c-Kit^+^ cells it is higher by about 16% (graph not shown). This result confirms that HIF-1α presence is stimulated in c-Kit^+^ cells. Indeed, the activated form of c-Kit, phosphorylated in Tyr719, is clearly more intense in c-Kit^+^ cells. 

### 2.4. C-Kit Positive hAFSCs and Total hAFSCs: Comparison of the Secretome

**Figure 5:**
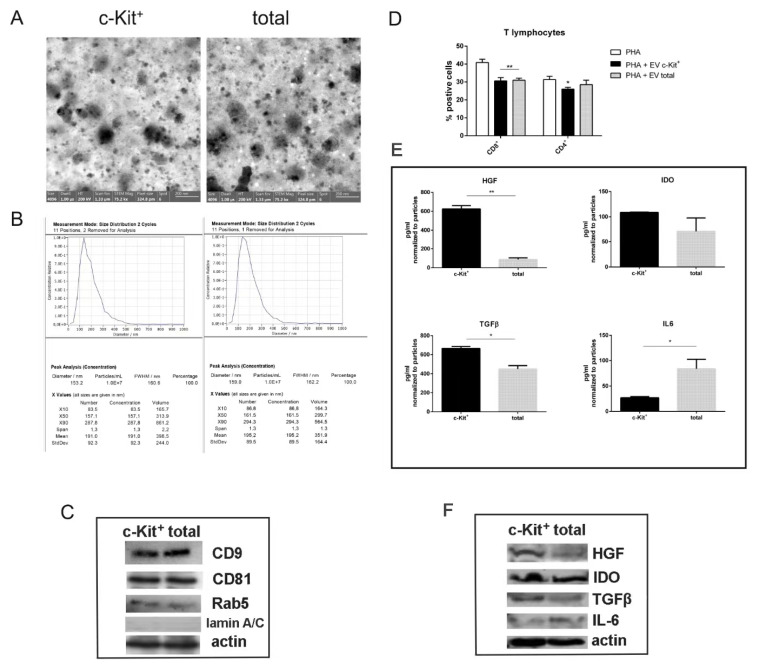
Comparison of the extracellular vesicles produced by amniotic fluid cells selected or not for c-Kit. (**A**) Representative TEM images of negative staining of vesicles obtained from two types of amniotic fluid cells. (**B**) Representative nanoparticle tracking analysis (NTA) performed on EV suspension with ZetaView. (**C**) Representative images of Western blot analysis of EV samples, revealed with anti-CD9, anti-CD81, anti-Rab5, anti-lamin A/C, and anti-actin as a loading control. (**D**) PBMCs, treated with PHA, were exposed for four days to EVs. The graphs show the cytofluorimetric analysis of the percentage of PBMC positive for CD4 and CD8 surface markers. ** *p* < 0.01 = samples significantly different. (**E**) ELISA analysis of TGFβ1, IDO, HGF, and IL-6 on the two lysates of isolated EVs. ** *p* < 0.01; * *p* < 0.05 = samples significantly different. (**F**) Representative images of Western blot analysis of EV samples, revealed with anti-HGF, anti-IDO, anti-TGFβ, anti-IL-6, and anti-actin, as a loading con.trol.

We compared the vesicular part of secretomes obtained from c-Kit^+^ and total cells. At first, the conditioned medium (CM) was collected and EVs were isolated, as described in the Method section. Transmission electron microscopy (TEM) analysis was performed in order to measure the dimensions of isolated vesicles. Figure 5A shows representative images of the samples of EVs isolated from CM. The median diameter of vesicles was around 100 nm. Moreover, the images of Figure 5B show the Nanoparticle Tracking Analysis (NTA) analysis performed on hAFSC-EVs: comparing the samples, the measures were similar, since the average of the median value of diameter particles of c-Kit positive cells was 160.1 ± 4.5 nm, while the one of total cells was 163.5 ± 5.5 nm. The number of vesicles obtained from CM deriving from c-Kit^+^ and from total was similar at around 1.5 × 10^9^ ± 0.50 from 1 × 10^6^ hAFSCs. The expression of typical EV markers, as CD9, CD81, and Rab5, was similar as well and the absence of lamin A/C confirmed the lack of contaminations, at least of nuclear components, in EVs. Then, activated PBMC were exposed to EVs for four days. The analysis for CD4, CD8, for T lymphocytes, showed that the EVs produced by c-Kit^+^ cells and by total cell population reduced in a similar manner both the T lymphocytes (Figure 5C).

Finally, we tested the quantity of TGF, HGF, IDO, and IL-6 into the EVs obtained from CM of the two cell types. Interestingly, as shown in graphs in Figure 5D and in WB images, the anti-inflammatory and pro-regenerative molecules, namely TGFβ1, HGF, and IDO [17], were more expressed into EVs derived from c-Kit^+^ cells, unlike the pro-inflammatory factor IL-6.

## 3. Discussion

In the field of regenerative medicine, although embryonic stem cells (ESCs) and induced pluripotent stem cells (iPSCs) should be the best sources of stem cells, their use in clinical practice raises ethical and practical concerns, since, for example, they can be tumorigenic. On the other hand, Perinatal SCs, such as amniotic fluid cells, can be easily isolated during routine medical procedures (amniocentesis) or at the delivery (during caesarium). These young cells maintain a broadly multipotent phenotype similar to the ones of ESCs and iPSCs since they have the capacity to differentiate into cells of all three embryonic germ layers, but they are not tumorigenic and have a low immunogenicity, being weakly positive for the major histocompatibility class II molecules [18]. These properties and the promising outcomes obtained from preclinical studies make hAFSCs a good alternative to ESC and iPSCs in regenerative medicine applications, however reports regarding clinical trials using adherent hAFSC alone are still lacking. However, generally speaking and in regards to perinatal stromal cells, multiple phase I/II clinical trials have confirmed the safety of perinatal MSC administration in regenerative medicine protocols and in the treatment of various chronic diseases. This delay on hAFSC application could be due to the fact that stem cells suspended in the amniotic fluid form a heterogeneous group of cells with distinct properties and cellular characteristics, including CD34^+^ hematopoietic progenitors, c-Kit^−^ mesenchymal stem/stromal cells (MSCs), and c-Kit^+^ multipotent stem cells [19]. While many laboratories are working with c-Kit^+^ isolated amniotic cells, others do not select this subpopulation. Each of these amniotic fluid cell types possesses its own unique tissue-specific characteristics that lends themselves to potential clinical translation based on differentiation capacity, anti-inflammatory effects, and other trophic properties that are known to promote tissue repair and regeneration. Despite these results, no definitive experiments have been provided for the analysis of “stemness features” of the different populations of hAFSCs.

In our study, we attempted to delineate some biological characteristics of the hAFSCs derived from the isolation process vs. the total amniotic fluid cell population by using single cell analysis coupled with RNA bulk. Although the selection process involved cell expansion, which led to a decrease in the c-Kit marker itself, the main findings of this study are that: (1) c-Kit^+^ hAFSCs expressed higher levels of pluripotency markers; (2) The capacity of modulating oxidative stress of c-Kit^+^ hAFSCs and their mitochondrial profile suggests that these populations can perform mitochondrial metabolism maintaining a redox balance; (#) Their secretome, namely the EV part, could be more effective than the one secreted by total amniotic fluid cells in promoting the remodelling of damaged tissue microenvironments.

Transcriptome analysis revealed that the selection process did not affect the number of distinguishable clusters, rather modifying the cellular distribution among them. Results of pathways and biological functions analyses performed on cluster-specific markers brought to light, among other things, an upregulation in c-Kit^+^ enriched clusters of HIF-1α signaling, oxidative phosphorylation, and stem cell proliferation, which are all pathways known to be involved in regulating pluripotency and proliferation of stem cells [20,21]. 

Actually, the enrichment in the c-Kit^+^ (stem cell factor receptor) population obtained by magnetic cell sorting (Figure 3A) and the production of SCF, its ligand, (Figure 1A, B), suggests an autocrine process in this subpopulation: in fact, the higher presence of the receptor is coupled by its activation, namely the tyrosine phosphorylation. Interestingly, the hypoxia inducible factor itself (HIF-1α) was more expressed in these cells, as suggested also by transcriptome data, even though we were culturing them in normoxia. In the literature, two hypotheses have been proposed as involved in this phenomenon: under normoxic conditions, SCF/c-Kit binding increases expression of HIF-1α through the PI3K/Akt and Ras/MEK/ERK pathways, even if in an experimental in vitro model of cancer [22]. The other one, which is in MSC, reported that the overexpression of HIF-1α in normoxia increased production of one of the most important hematopoietic growth factors, SCF [23]. 

Even if we cannot demonstrate which of them is the one involved in this network, HIF-1α regulates cellular metabolism that holds numerous downstream target genes and interacts with other signaling pathways. Indeed, this factor modulates different metabolic processes including oxidative stress responses, glycolysis, and mitochondrial respiration [24]. However, how the HIF family is involved in the maintenance of SC pluripotency is still under debate. For example, Piccoli et al. [25] demonstrated that NADPHoxidase-dependent ROS production controls the stabilization of HIF-1α levels in circulating hematopoietic stem cells, suggesting that, even in normoxia, SCs could maintain anaerobic metabolism. They proposed a link between HIF-1α and ROS to the SCs self-renewal capacity under normoxic conditions. On the other hand, other authors have reported that ROS increased HIF-1α expression [26]. Here, we demonstrated that ROS levels were higher in c-Kit^+^ samples, but this event could be the side effect of the mitochondrial activity that should be more intense, since these cells show a bigger mitochondrial area (footprint and signal). However, the upregulation of antioxidant enzymes and/or pathways occurring in c-Kit^+^ samples (heatmap in Supplementary Figure S1) shows a cell defense response that could lead to an intracellular redox balance. To sum up these findings, our results on the interplay between ROS, the regulation of HIF stability, and hAFSC pluripotency suggest that c-Kit^+^ cells can better proliferate in vitro while still maintaining plasticity. 

These findings are consistent with our previous study [7] on the subpopulations present in hAFSC, which showed that a higher proliferative potential, improved replicative lifespan, and enhanced expression of embryonic stem cells markers Oct4 and SSEA-4 were upregulating the ES pathway and the Hypoxia pathway, which in turn suggested an altered metabolic profile and increased mitochondrial biogenesis and activity. 

Progenitors, typically characterized by high self-renewing and proliferative processes, also use an active mitochondrial metabolism [27]. Therefore, the general idea that stem cells, as examples of glycolytic cells, have fragmented mitochondria, is not always correct. In fact, by looking at different stem cell types and at cancer cells, it seems that the link between proliferation, mitochondrial metabolism, and mitochondrial dynamics is not well defined and probably highly plastic and environment-dependent. From the evaluation of the transcriptome profiles of the two cell populations shown in Figure 1 and Figure 2, we can see an upregulation of oxidative phosphorylation for c-Kit^+^ cells compared to total samples, and the opposite trend for glycolytic metabolism.

Here we noticed that the mitochondrial morphology was different when comparing the total AF cells with the c-Kit^+^ ones: mitochondria of the latter population appeared fused and image analysis demonstrated that the dimension, unlike the number, was significantly higher than in total AF cells. Mitochondrial fusion was mainly mediated by optic atrophy 1 (Opa1) located on the mitochondrial outer membrane and mitofusin 1/2 (Mfn1/2) located on the inner membrane [28]. Indeed, here we demonstrated with single cell analysis that these genes are upregulated in several clusters of c-Kit^+^ cells.

Notably, mitochondrial dynamics regulate cell signaling processes involved in the maintenance of stemness of several stem cell populations. In embryonic stem cells (ESCs), mitochondrial fusion, regulated by the outer mitochondrial membrane protein MTCH2, is a key regulator in the naïve-to-primed pluripotency interconversion [29]. It is known that mitochondrial dynamics can be modified by extracellular/intracellular stimuli such as reactive oxygen species and redox signaling, nutrient availability, as well as different growth factors. Mitochondrial fusion may occur as a compensatory event in response to weak stress, for example to be cultured in normoxia and not in hypoxic physiological oxygen tension as in our experimental conditions, to promote survival. In many cases, mitochondrial elongation is beneficial for counteracting stress [30,31]. 

MSCs exert their therapeutic effects largely through their paracrine actions. The effects of MSC-sourced secretomes derive from their capacity to transfer genetic material, growth, and immunomodulatory factors to the target cells, thus regulating anti-apoptotic and pro-survival pathways that can be benficial in stimulating tissue repair and regeneration. Growth factors, cytokines, chemokines, extracellular matrix components, and metabolic products were all proteins found to be functional molecules of MSCs in various therapeutic paradigms [32]. Among MSC-derived immunomodulatory and trophic factors, IDO, TGF-β, and HGF contributed to the beneficial effects of MSC-secretome even in its vesicle part. We analyzed extracellular vesicles with dimensions around 160 nm, being aware that we could not distinguish between exosomes and microvesicles. The observation that the EV content in the factors, able to remodel damaged tissue microenvironment such as TGF-β, and HGF [33], was higher in c-Kit^+^ cells, if compared to the total population of hAFSCs, sustains the hypothesis that the paracrine effect of c-Kit^+^ cells can better contribute to tissue remodelling and cellular homeostasis during regeneration.

## 4. Materials and Methods

### 4.1. Amniotic Fluid Collection

The hAFSCs were obtained from 3 amniotic fluids (AF) collected from pregnant women (mean age 36.2 ± SD 4.0) between the 16th and 17th weeks of gestation who underwent amniocentesis for maternal request (not for foetal anomalies) at the Unit of Obstetrics & Gynecology, at the Policlinico Hospital of Modena (Italy). The collection from amniocentesis was performed as previously reported [17] (protocol 360/2017 dated 12.15.2017 approved by Area Vasta Emilia Nord).

### 4.2. Human Amniotic Fluid Cell Culture

For this study, supernumerary (unused) flasks of adherent AF cells cultured in the Laboratory of Genetics of TEST Lab for 2 weeks in Amniochrome^TM^ -II complete medium (Lonza Bioscience, Rome, Italy) were used. Cells from each donor were processed separately. After trypsinization, cells were expanded until the 3rd passage in culture in culture medium (αMEM) supplemented with 20% foetal bovine serum (FBS), 2 mM L-glutamine, 100 U/mL penicillin, and 100 μg/mL streptomycin (all from EuroClone Spa, Milano, Italy) in order to reach at least 2 × 10^6^ cells. They were then divided into two groups:

- one (total) was not manipulated anymore and frozen;

- the other one (c-Kit^+^) was obtained after the isolation of the c-Kit positive population.

Cells subjected to c-Kit immunoselection by MACS technology (Miltenyi Biotec, Germany) and selected from at least 1.5 × 10^6^ cells represent 1–4% of the processed sample. Both hAFS cell groups were subcultured routinely at 1:3 dilution and not allowed to grow beyond the 70% of confluence in 75 cm^2^ flasks (around 1 × 10^6^ cells). Then, an expansion procedure occurred, and we performed a comparison between total and c-Kit^+^ at the same passage in culture for each donor.

### 4.3. Bulk RNA Transcription Profiling

Total RNA was extracted using the RNeasy Mini Kit (Qiagen) following the manufacturer’s instructions. Libraries were prepared using TruSeq Stranded Total RNA Library Prep Gold kit (Illumina, San Diego, CA, USA, Cat #: 20020599) and sequencing was performed on NovaSeqTM 6000 (Illumina) running in a 50-bp pair-end mode. After quality control performed with FASTQC, reads were trimmed with TrimGalore v0.6.6 retaining reads with a quality score ≥ 20 and a read length ≥ 40 (https://www.bioinformatics.babraham.ac.uk/projects/trim_galore/, accessed on 20 January 2023). The filtered reads were mapped on the human genome (GRCh38/hg38) using STAR aligner with the option --quantMode GeneCounts [34]. Images of selected genes were saved using bgwg files loaded into IGV_2.11.0 software. Enrichment analysis was obtained with the software Gene Set Enrichment Analysis (GSEA 4.2.1) (http://software.broadinstitute.org/gsea/index.jsp, accessed on 20 January 2023) and performed using TPM values, removing genes with an average RPKM value between the two conditions less than 1. Here the hallmark collection was used together with the stemness signature, the list “ES exp1”, reported in Ben-Porath et al. [35]. Just terms with FDR < 0.05 were graphed.

### 4.4. Single Cell RNA-seq and Bioinformatic Analysis

5000 cells were isolated and library prepared using the Chromium Next GEM Single Cell 3′ GEM, Library & Gel Bead Kit v3.1 on 10X genomics platform and sequenced on NovaSeqTM 6000 (Illumina) with a coverage of 50,000 reads/ cell. Alignment and count matrix generation was performed using Cell Ranger v6.0.1 on hg38. Downstream analysis was conducted using the R package Seurat v4.1.0 [36]. In brief, cells nFeature_RNA > 200 and nFeature_RNA < 7000 & percent.mt < 5 were retained, then the datasets were normalized with SCT v2 method and merged, and clusterization was performed. FindMarkers function with “MAST” test was used to characterize specific markers of each cluster. Markers with a logFC > |0.5| were used to perform Ingenuity Pathway Analysis. Pathways and functions with an absolute z-score of >2 were considered significant. 

### 4.5. Cell Cycle Analysis

The assay was carried out by the Muse™ Cell Cycle kit (MCH100106; Merck Millipore, Billerica, MA, USA) to analyze the amounts of DNA at cell cycle stages (sub-G1, G0/G1, S and G2/M). The hAFSC (8×10^4^ cells/well) were plated in 6-well plates and the next day the cells were harvested and suspended in 1× PBS, and then fixed in cold 70% ethanol overnight at −20 °C. The fixed cells were centrifuged and stained with Muse™ Cell Cycle kit reagent in the dark for 30 min at room temperature. Cell cycle distribution was measured by a Muse™ Cell Analyzer.

### 4.6. Immunofluorescence and Confocal Microscopy

For immunofluorescence analysis, total and c-Kit^+^ hAFSCs were processed and confocal imaging was performed using a Nikon A1 confocal laser scanning microscope.

Primary antibodies to detect Oct4, c-Kit, SSEA-4 (Cell Signaling, MA, USA), h-mitochondria (h-mit) (Merck Millipore, MA, USA), and TRA1-81 (Santa Cruz Biotechnology, CA, USA) were used following datasheet recommended dilutions. Alexa secondary antibodies (Thermo Fisher Scientific, Waltham, MA, USA) were used at 1:200 dilution.

Image elaboration was performed as previously reported [7].

### 4.7. Mitochondria Analysis

Mitochondrial analyses were performed using ImageJ plug-in Mitochondria Network Analysis tool (MiNA) as previously described by Valente et al. [37]. Briefly, confocal images labelled with h-mitochondria (h-mit) (Merck Millipore, MA, USA) were pre-processed using unsharp mask, CLAHE, and median filter to enhance image quality. Then, skeletonized images, obtained with the “Skeletonize” ImageJ feature, were analyzed by MiNA plug-in. 

### 4.8. ROS Detection

To evaluate the intracellular ROS levels, a dichlorodihydrofluorescein diacetate (DCFH-DA) assay was performed as previously described [38]. The hAFSCs were seeded into 96-well plates with 5 replicates for each condition. The next day, culture medium was removed from each well and 5 µM DCFH-DA or MitoTrakerTM were incubated in PBS with 1 gr/l of glucose for 20 min for 10 min at 37 °C and 5% CO_2_. The probe solution was replaced with PBS/glucose and the fluorescence was read at 485 nm (excitation) and 535 nm (emission) for DCFH-DA and MitoTrackerTM using the multiwell reader Appliskan (Thermo Fisher Scientific, Waltham, MA, USA). Cellular autofluorescence was subtracted as a background using the values of the wells not incubated with the probe.

### 4.9. Extracellular Vesicle Isolation from Conditioned Medium

Before extracellular vesicle extraction, the cells grown in 75 cm^2^ flasks (around 1 × 10^6^ cells) were washed with PBS and maintained for 4 days in 10 mL culture medium deprived of FBS in order to exclude the contamination by extracellular vesicles included into the FBS solution. The secreted part of the conditioned medium (CM) was then concentrated up to 2 mL using Centrifugal Filter Units with 3K cutoff.

CM were centrifuged at 10,000× *g* for 30 min at 4 °C. The supernatants transferred to poly(propylene) ultracentrifuge tubes (13.2 mL, Beckman Coulter) were centrifuged at 100,000× *g* for 90 min at 4 °C in a Beckman Coulter Optima L-90K centrifuge with a SW-41 rotor. The supernatant was discarded while pellets were washed in PBS and centrifuged again. The pellet was resuspended into 100 μL of PBS. 

### 4.10. NTA Analysis

Nanoparticle tracking analysis (NTA): EVs were diluted 1:500 in PBS and visualized by ZetaView particle tracker from ParticleMetrix (Inning am Ammersee, Germany). Five recordings (60 s) were performed. NTA software provided high-resolution particle size distribution profiles and concentration measurements.

### 4.11. EV Characterization by Transmission Electron Microscopy

Transmission electron microscopy (TEM) assays were performed as previously reported [17]. Briefly, exosome pellets were suspended in and fixed with 4% paraformaldehyde and 4% glutaraldehyde in 0.1 M phosphate buffer (pH 7.4) at incubation temperature and kept at 4 °C until analysis. A drop of each exosome sample was placed on a carbon-coated copper grid and immersed in 2% phosphotungstic acid solution (pH 7.0) for 30 s. The preparations were examined with a transmission electron microscope (JEM-1200EX, JEOL Ltd., ToKyo, Japan) at an acceleration voltage of 80 kV.

### 4.12. Protein Quantification in Purified EVs

After centrifugation, samples from EV fractions were analyzed using a NanoDrop ND-1000 spectrophotometer (Thermo Fisher Scientific, Waltham, MA, USA) with the Protein A280 protocol. The concentration was related with NTA data to obtain the number of EVs/µg protein value.

### 4.13. ELISA Assays

Briefly, EVs were lysed by treating with a lysis buffer at a ratio of 1:3 (vol:vol) followed by 3 cycles of freeze and thaw. Concentrations of HGF, TGFβ, IDO, IL-6, and HIF-1α in the EVs of total or c-Kit^+^ hAFSCs were measured by using ELISA assays, according to the manufacturer’s instructions (Boster Biological Technology, Pleasanton, CA; MyBioSource, Peachtree Corners, GA). Samples were run in duplicate. A standard curve was constructed using known concentrations of recombinant human standards.

### 4.14. PBMC Exposure to hAFSC EVs: Flow Cytometer Immune-Assay

Human peripheral blood mononuclear cells (PBMCs) were separated from the peripheral blood of healthy donors by gradient centrifugation on Ficoll-Hypaque (Lymphoprep, AXIS-SHIELD PoCAs, Oslo, Norway) at room temperature (RT). The concentration of isolated PBMC was adjusted to 2 × 10^6^ cells/mL in RPMI 1640 including 10% FBS, 2 mM L-glutamine, 100 U/mL penicillin, and 100 μg/mL streptomycin (all from EuroClone Spa, Milano, Italy). Twenty hours later, PBMCs were washed, and used for experiments described below.

PBMCs in the majority of experiments were activated with 5 µg/mL phytohemoagglutinin (PHA) for 24 h prior to and during the phase of EV exposure. A volume corresponding to 8µg of proteins of EVs was added to the PBMC suspension (2 × 10^5^ cells/200µL) for 4 days.

PBMCs were stained with the following kits: Muse™ Human CD8 T Cell Kit and Muse™ Human CD4 T Cell Kit (MIM100102 and MIM100101, Merck Millipore, Billerica, MA, USA). A minimum of 10,000 cells per sample was acquired and analyzed using Muse™ Cell Analyzer.

### 4.15. Cellular and EV Extracts Preparation

Cell and EV extracts were obtained as previously described [17].

### 4.16. SDS PAGE and Western Blot

Whole cell lysates from hAFSC and hAFSC-EV were processed as previously described [39]. Primary antibodies were raised against the following molecules: HIF-1α, GAPDH, actin, HGF, IDO, lamin A/C (Santa Cruz Biotechnology, CA, USA), phospho-c-Kit^Tyr719^ (Cell Signaling Technology, MA, USA), TGFβ (Novus, CO, USA), Rab5 (Lonza, SC, USA), CD9, and CD81 (Life Technologies, CA, USA).

Secondary antibodies, used at 1:3000 dilution, were all from Thermo Fisher Scientific (Waltham, MA, USA).

### 4.17. Statistical Analysis

Three replicates were performed for in vitro experiments. Values were reported as mean ± SE based on triplicate analysis for each sample. Student’s t-test or One-way Anova with Bonferroni post hoc test were applied. The level of significance was set at *p* value < 0.05. Statistical analysis was obtained by using GraphPad Prism^®^ release 6.0 software.

## Data Availability

RNA-seq data are deposited on GEO repository and accessible with GSE219026 number (https://www.ncbi.nlm.nih.gov/geo, accessed on 20 January 2023). All the data that support the figures and the other findings are available from the authors upon request.

## Supplementary Materials

The following supporting information can be downloaded at: https://www.mdpi.com/article/10.3390/biomedicines11020430/s1, Figure S1: Heatmap of genes in gene set belonging to Reactive Oxygen Species Pathway; Figure S2: Heatmap showing the RNA expression level of Fusion-Fission mitochondrial genes.
