# Supplementary material for: Effect of the Enrichment in c-Kit Stem Cell Potential of Foetal Human Amniotic Fluid Cells: Characterization from Single Cell Analysis to the Secretome Content
Source: Biomedicines. 2023 Feb 2;11(2):430. doi: 10.3390/biomedicines11020430 (PMC9953071; doi:10.3390/biomedicines11020430)
Supplement: Supplementary file 1 [file biomedicines-11-00430-s001.zip › biomedicines-2154229-supplementary.pdf]

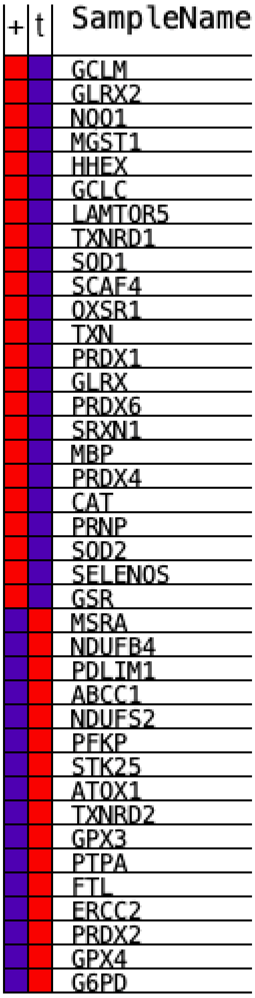

Supplementary Figure S1. Heatmap of genes in gene set belonging to Reactive Oxygen Species Pathway (GSEA analysis). Lowest level of expression is represented by blue, highest level in red.

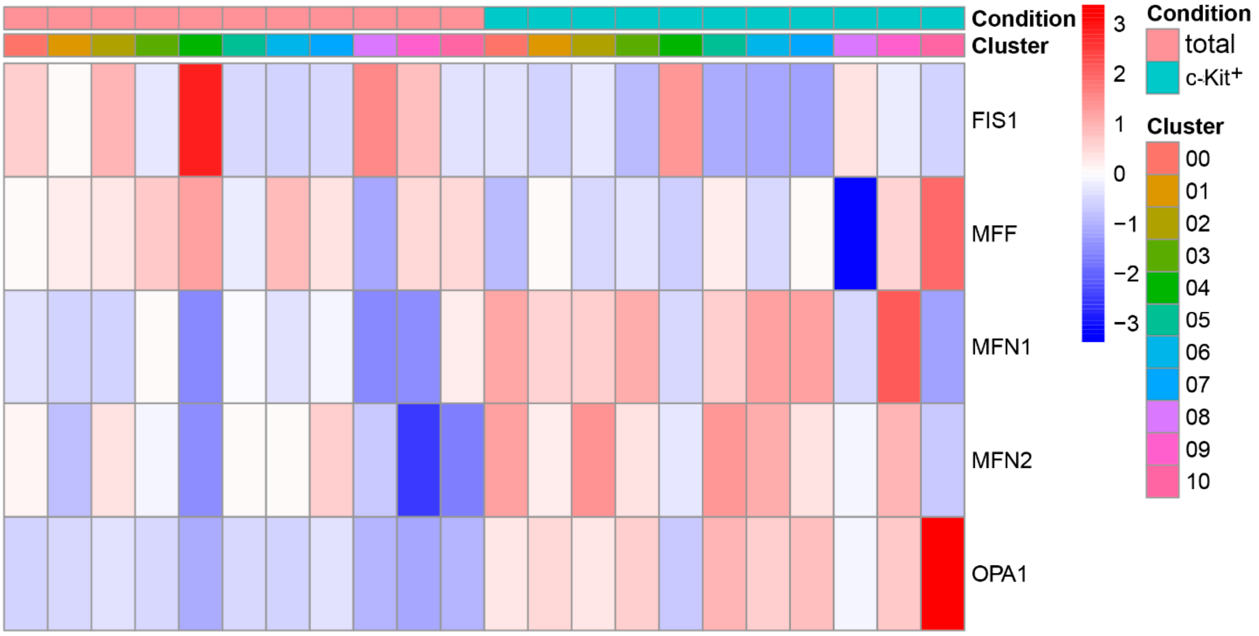

Supplementary Figure S2. Heatmap showing the RNA expression level of Fusion-Fission mitochondrial genes in the single-cell identified clusters both in total and c-Kit+ sample. Lowest level of expression is represented by blue, highest level in red.
